# Supplementary material for: Bacteriocin Production by Escherichia coli during Biofilm Development
Source: Foods. 2022 Sep 1;11(17):2652. doi: 10.3390/foods11172652 (PMC9455227; doi:10.3390/foods11172652)
Supplement: Supplementary file 1 [file foods-11-02652-s001.zip › foods-1863955-supplementary.pdf]

**Table S1**. Bacterial strains used in this study

| Strain | Serotype | Source | Verotoxin production |
|---|---|---|---|
| ***E. coli* CECT 4782** | O157:H7 | Human stool from the outbreak of hemorrhagic colitis | stx1, stx2 |
| ***E. coli* CECT 4783** | O157:H7 | Raw hamburger meat implicated in hemorrhagic colitis outbreak | stx1, stx2 |
| ***E. coli* CECT 4267** | O157:H7 | Human stool from the outbreak of hemorrhagic colitis | stx1, stx2 |
| ***E. coli* CECT 5947** | O157:H7 | - | Gene stx2 has been replaced |
| ***E. coli* NCTC 12900** | O157:H7 | - | NT |
| ***E. coli* CECT 352** | O127a:K63(B8):H- | - | EPEC |
| ***E. coli* CECT 504** | O141:K85(B):H4 | Swine edema | ND |
| ***E. coli* CECT 515T** | O1:K1(L1):H7 | Human urine -cystitis | ND |
| ***E. coli* CECT 533** | O103:K-:H- | - | ND |
| ***E. coli* CECT 727** | O111:K58(B4):H- | Infantile gastroenteritis | EPEC |
| ***E. coli* CECT 730** | O55:K59(B5):H- | - | ND |
| ***E. coli* CECT 736** | O28a,28c:K73(B18):H- | Faeces | ND |
| ***E. coli* CECT 740** | O125a,125b:K70(B15):H19 | Human gastroenteritis | ND |
| ***E. coli* CECT 744** | O158:K-:h23 | Faeces of an infant with diarrhea | ND |
| ***E. coli* CECT 832** | O111:K58(B4):H- | Infantile gastroenteritis | ND |
| ***E. coli* CECT 4537** | O10:K5(L5):H4 | Human peritonitis | ND |
| ***E. coli* CECT 4555** | O97:K-:H- | - | ND |
| ***E. coli* CECT 434** | O6:KN | Human gastroenteritis | ND |
| ***E. coli* N5*** | ND | Bovine faeces | ND |
| ***E. coli* ATCC 29425 (K12)** | OR:H48:K- | - | ND |
| ***E. coli* K12 Δ*impA*** | - | Laboratory collection | - |
| ***E. coli* ER1100A Δ*entF*** | - | Laboratory collection | - |
| ***Listeria monocytogenes* CECT 5873** | - | - | - |
| ***Salmonella enterica* SGSC 2476** | - | - | - |
| ***Shigella dysenteriae* ATCC 11335** | - | - | - |
| ***Staphylococcus aureus* CECT 86** | - | - | - |
| ***Staphylococcus epidermidis* CECT 4184** | - | - | - |

NT – non-toxigenic *E. coli*; EPEC – enteropathogenic *E. coli* (epidemiologically implicated as pathogens, but virulence mechanism is not related to the excretion of enterotoxins); ND – Not determined; * - Isolates; SGSC – *Salmonella* Genetic Stock Centre; ATCC – American Type Culture Collection; NCTC - National Collection of Type Cultures; CECT - Spanish Type Culture Collection.

**Table S2**. Primers used in this study

| Target colicin gene | Primers sequence (5′ to 3′) | | Tm (°C) | Amplicon size (bp) |
|---|---|---|---|---|
| **For PCR studies** | | | | |
| Ia; Ib | Fw | GCAGACACGGAATGACAGGGC | 68 | 406 |
| | Rv | GCCGTAACTTATCCCATTCAGC | 66 | |
| E1-E3; E6; E8-E9 | Fw | CCTTATGATGATAAGGGGCAGG | 66 | 576 |
| | Rv | CCAGCTCAGATTGTGCAGCAGC | 70 | |
| E7 | Fw | CCGAGAACCAATGGCTGCTGG | 68 | 476 |
| | Rv | CCTGGGTTCTAGTCTTGGGCG | 68 | |
| 5; 10 | Fw | CCAGAGTTGCAGGRGAGC | 59 | 658 |
| | Rv | CATTAATGCCACAATTTTTGCC | 60 | |
| B | Fw | CCAAAGGCTATAAGGGCCGAGC | 70 | 709 |
| | Rv | CCCGAAATCCAGGAAGATGGCG | 70 | |
| M | Fw | GGTTACTTCTGTAACGCCG | 58 | 495 |
| | Rv | GCCTTGTGAGCGACTCTCC | 62 | |
| **For qPCR studies** | | | | |
| B | Fw | CCACACTTAATACCAGGTCCGG | 57 | 124 |
| | Rv | CCGATGACAGTGCCAGTAGTGG | 59 | |
| M | Fw | CCAAACATGTGTCTTCAGGC | 52 | 140 |
| | Rv | GGGTGAAGAACCAGATTTCG | 52 | |
| E | Fw | GGGCGCGCATAGCACAAGTGG | 60 | 124 |
| | Rv | GCTACCGGAACCACCACCC | 58 | |
| GAPDH | Fw | GCCTCTTTTTCGGCGTAAACC | 54 | 129 |
| | Rv | CCCCAAAAATTCTCGCCTACC | 54 | |

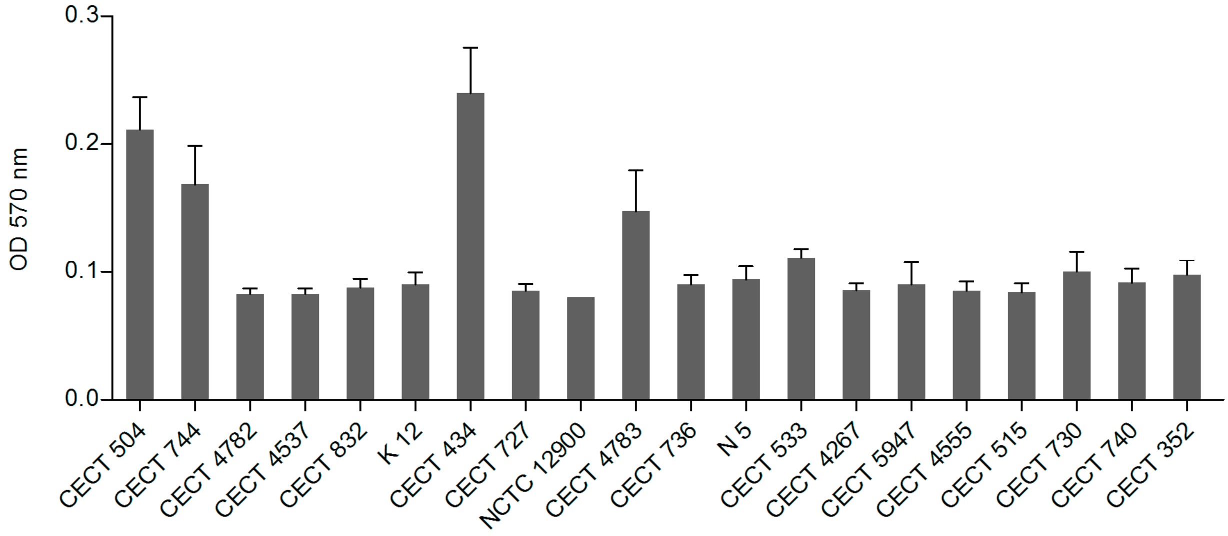


**Figure S1.** Single-species biofilm formation of *E. coli*. Biofilms were grown in 96-well microtiter plates for 24 h in LB and LB supplemented with 0.25 % (w/v) glucose media. The biomass amount was assessed by the crystal violet method as described in the methods section of this manuscript.
